# Phylogeny and Biogeographic History of *Parnassius* Butterflies (Papilionidae: Parnassiinae) Reveal Their Origin and Deep Diversification in West China

**DOI:** 10.3390/insects13050406

**Published:** 2022-04-23

**Authors:** Youjie Zhao, Bo He, Ruisong Tao, Chengyong Su, Junye Ma, Jiasheng Hao, Qun Yang

**Affiliations:** 1 College of Life Sciences, Anhui Normal University, Wuhu 241000, China; bioala@ahnu.edu.cn (Y.Z.); hebo90@126.com (B.H.); sky475342@163.com (C.S.); 2 State Key Laboratory of Palaeobiology and Stratigraphy, Center for Excellence in Life and Palaeoenvironment, Nanjing Institute of Geology and Paleontology, Chinese Academy of Sciences, Nanjing 210008, China; jyma@nigpas.ac.cn; 3 College of Life Sciences, Hefei Normal University, Hefei 230001, China; trs.cm@163.com; 4 Nanjing College, University of Chinese Academy of Sciences, Nanjing 211135, China

**Keywords:** genus *Parnassius*, mitochondrial genes, biogeographic history, phylogenetic network, Qinghai–Tibet Plateau

## Abstract

**Simple Summary:**

Butterflies of the genus *Parnassius* are distributed in the mountains across the Northern Hemisphere. Studies have shown that this genus originated in the regions of West China–Central Asia. To further explore the spatiotemporal pattern and driving mechanism of *Parnassius* diversification, we reconstructed the phylogeny and biogeographic history of *Parnassius* based on 45 species. Ancestral area reconstruction obtained by using the statistical dispersal–extinction cladogenesis model indicated that *Parnassius* originated in West China (Qinghai–Tibet Plateau and Xinjiang) during the Middle Miocene. Paleoenvironment changes and host plants were probably influenced by the dispersal of *Parnassius* butterflies from West China to East Asia, Europe, and North America. Furthermore, ancient gene introgression might have contributed to the spread of *Parnassius* butterflies from the high mountains of the Qinghai–Tibet Plateau to the low-altitude areas of Central East China. This study will provide an understanding of the phylogeny and biogeographic history of the genus *Parnassius*.

**Abstract:**

We studied 239 imagoes of 12 *Parnassius* species collected from the mountains of the Qinghai–Tibet Plateau (QTP) and its neighbouring areas in China. We selected three mitochondrial gene (*COI*, *ND1*, and *ND5*) sequences, along with the homologous gene sequences of other *Parnassius* species from GenBank, to reconstruct the phylogenetic tree and biogeographic history of this genus. Our results show that *Parnassius* comprises eight monophyletic subgenera, with subgenus *Parnassius* at the basal position; the genus crown group originated during the Middle Miocene (ca. 16.99 Ma), and species diversification continued during sustained cooling phases after the Middle Miocene Climate Optimum (MMCO) when the QTP and its neighbouring regions experienced rapid uplift and extensive orogeny. A phylogenetic network analysis based on transcriptomes from GenBank suggests that ancient gene introgression might have contributed to the spread of the *Parnassius* genus to different altitudes. Ancestral area reconstruction indicates that *Parnassius* most likely originated in West China (QTP and Xinjiang) and then spread to America in two dispersal events as subgenera *Driopa* and *Parnassius*, along with their host plants Papaveraceae and Crassulaceae, respectively. Our study suggests that extensive mountain-building processes led to habitat fragmentation in the QTP, leading to the early diversification of *Parnassius*, and climate cooling after MMCO was the driving mechanism for the dispersal of *Parnassius* butterflies from West China to East Asia, Europe, and North America.

## 1. Introduction

Geologic and paleoclimatic events have played crucial roles in the origin and evolution of biodiversity on Earth. The collision of the Indian and Eurasian plates, followed by the uplift of the Qinghai–Tibet Plateau (QTP) [1,2,3] and subsequent sharp climate changes [4,5,6] during the Cenozoic are considered the major driving forces of Asian biodiversity [7,8,9]. *Parnassius* Latreille,1804 butterflies are commonly distributed in high-altitude mountains across Asia, Europe, and North America [10], with the highest diversity concentrated in the QTP and neighbouring areas [11,12,13,14]. *Parnassius* is the most species-rich genus in the subfamily Parnassiinae Duponchel, 1835 (Lepidoptera and Papilionidae), with approximately 55 extant species. These butterflies are highly sensitive to environmental change, and thus, their phylogeographical structure is considered to reflect climate-driven range shifts; their geographic ranges or connectivity expands during glacial periods, whereas it reduces during interglacial periods due to the interruption of the gene flow [15,16]. *Parnassius* has recently attracted much attention as a model organism of alpine invertebrates in the investigation of climate change effects on organisms in the Northern Hemisphere [17,18].

Studies have shown that the early *Parnassius* phylogenies, based on morphology (wing pattern, venation, or male genitalia), split the genus into up to 10 lineages or subgenera [19,20,21,22,23,24]. Later on, the evaluation of mitochondrial or nuclear genes (*ND1*, *ND5*, *16S*, *COI*, *LSU*, *EF-1α*, or *wg*) revealed that *Parnassius* species are clustered into eight subgenera—that is, *Parnassius* Latreille, 1804, *Driopa* Korshunov, 1988, *Tadumia* Moore, 1902, *Lingamius* Bryk, 1935, *Kailasius* Moore, 1902, *Koramius* Moore, 1902, *Sachaia* Korshunov, 1988, and *Kreizbergia* Korshunov, 1990 [12,13,25,26,27]. Furthermore, this genus was found to originate in the region of West China–Central Asia during the Miocene [26,28]. However, the spatiotemporal pattern and driving mechanism of biogeographic formation need further exploration for the genus *Parnassius*.

**Figure 1:**
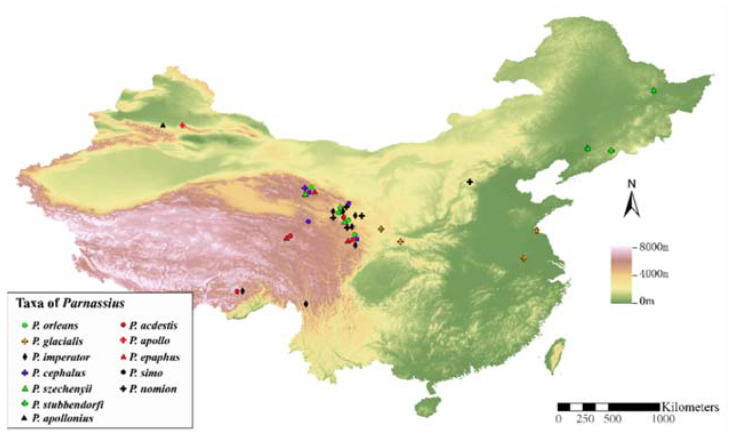
Sample locations of 12 *Parnassius* species collected in this study.

In this study, we collected numerous samples (239 imagoes of 12 *Parnassius* species) from different altitudes of the QTP and its neighbouring areas in China (Figure 1, Table 1, and Table S1). We used GenBank data (Table 1) to reconstruct the species-level phylogeny based on 45 species belonging to eight subgenera and analysed their historical biogeography, along with their paleogeographic events. In particular, we explored the potential mechanism of reticulate evolution in the formation of niches at different altitudes.

## 2. Materials and Methods

### 2.1. Specimen Collection

A total of 239 imagoes of 12 *Parnassius* species were collected from natural populations distributed in the QTP and its neighbouring areas (Figure 1, Table 1, and Table S1). The species were identified according to their morphological characteristics following Weiss and Rigout (2006) [10]. Fresh samples were immediately placed in 100% ethanol for fixation and preserved at −20 °C for subsequent experiments.

### 2.2. DNA Extraction, Polymerase Chain Reaction Amplification, and Sequencing

Total genomic DNA was extracted from the chest muscles of the samples by using a Rapid Animal Genomic DNA Isolation Kit (Sangon Biotech, Shanghai, China) following the manufacturer’s instructions. Three mitochondrial DNA segments (*ND1*, *ND5*, and *COI*) were amplified using primers reported in previous studies [29,30,31] (Table S2). All primers were synthesised by Sangon Biotechnology Co., Ltd., Shanghai, China. The polymerase chain reaction (PCR) procedures used the following cycling parameters: initial denaturation for 2 min at 94 °C; 35 cycles of 1 min at 94 °C, 1 min at 46–57 °C, and 1 min at 72 °C; and a final extension of 10 min at 72 °C. The PCR products were purified using a DNA Purification Kit (Tiangen Biotech, Beijing, China) and sequenced directly on an ABI 3730xl DNA analyser by General Biotechnology Co., Ltd., Wuhu, China.

The mitochondrial sequences (*COI*, *ND1*, and *ND5*) of 41 Parnassiinae species were downloaded from GenBank (Table 1). To reduce the effect of possible chimeras concatenated by the three mitochondrial segments, we selected the same or a neighbouring locality for each species. A total of 45 *Parnassius* species were studied, of which 12 species were newly sequenced and 37 species were from GenBank. *P. imperator*, *P. cephalus*, *P. epaphus*, and *P. nomion* came from not only GenBank but, also, this study.

### 2.3. Phylogenetic Analysis

The gene sequences were separately aligned using MUSCLE in MEGA6.0 [32] and concatenated into one dataset by using DAMBE7.0 [33]. Kimura 2-parameter distances were calculated using MEGA for all species and for the populations of each species. Haplotype diversity information was calculated using DnaSP v.5.0 [34]. Nei’s genetic distance matrix among populations and Mantel tests of relative contributions between genetic and geographic distances were estimated by GenALEx [35].

With four Parnassiinae species, namely *Luehdorfia taibai* Chou, 1994, *Luehdorfia chinensis* Leech, 1893, *Sericinus montelus* Gray, 1853, and *Hypermnestra helios* (Nickerl, 1846), as the outgroups, the phylogenies of 45 *Parnassius* species were reconstructed based on the sequence data of three mitochondrial genes (File S1) with the maximum likelihood (ML) and Bayesian inference (BI) methods. The ML analysis was conducted using IQ-TREE software, version 1.6.8, under the GTR + F + R4 models determined through ModelFinder [36], and the bootstrap value of each node of the ML tree was evaluated with 5000 replicates (-bb 5000 -m GTR + F + R4). The Bayesian analysis was performed using MrBayes 3.1.2 [37], and the best substitution model for each partition was selected as GTR + I + G under the Akaike information criterion by using Modeltest 3.06 [38]. The MCMC chains (with random starting trees) were run with one cold and three heated chains simultaneously for 1,000,000 generations and sampled at every 100 generations, with a burn-in of 25% samples discarded until the chain convergence was reached. The confidence value of each node of the BI tree is presented as the Bayesian posterior probability.

To verify the previous observation that *Parnassius* butterflies experienced early rapid radiation [39], we further analysed the reticulate evolutionary relationships based on the previously published transcriptomes of *Parnassius* (*P. imperator* Oberthür, 1883, *P. simo* Gray, 1852, *P. orleans* Oberthür, 1890, *P. acdestis* Grum-Grshimailo, 1891, *P. epaphus* Oberthür, 1879, *P. cephalus* Grum-Grshimailo, 1891, *P. glacialis* Butler, 1866, and *P. jacquemontii* Boisduval, 1836) and the outgroup species *S. montelus* [40]. High-quality reads were assembled using Trinity [41], and the obtained transcripts were clustered to unigenes by using Cd-hit-est (threshold 0.95) [42]; the putative orthologues were identified using OrthoFinder [43], and the single-copy unigenes were selected and aligned using MUSCLE [44] to obtain the information condensed by Gblock [45]. The maximum likelihood phylogenetic tree was reconstructed based on each orthologue with RaxML [46] by using *S. montelus* as the outgroup, and the phylogenetic networks were predicted with PhyloNet by using maximum pseudo-likelihood models under 0~2 reticulation [47].

### 2.4. Divergence Time Estimation

As time calibration priors, we used the oldest fossil of the subfamily Parnassiinae, namely *Thaites ruminiana* Scudder, 1875, from the Chattian Stage (23.03–28.1 Ma, Late Oligocene) [48] for the minimum age of the crown group Parnasiinae, with a lognormal distribution, and *Doritites bosniaskii* Rebel, 1898 from the Messinian (5.33–7.25 Ma, Late Miocene) of Italy (Tuscany) [48], as sister to *Archon* (Luehdorfiini), also constraining the crown of Luehdorfiini + Zerynthiini with this minimum age (5.33 Ma) in a lognormal distribution. The maximum bound of the calibration priors was set to 140 Ma based on the estimated age of the host–plant Angiosperms [49,50].

On the basis of the time priors, the divergence time of *Parnassius* was estimated using BEAST v1.83 [51]. The MCMC chain was run for 10 million generations to achieve convergence and was sampled every 1000 generations. Convergence was assessed from the effective sampling size after 10% of the burn-in samples were discarded using Tracer v1.6 [52]. The Maximum Clade Credibility (MCC) tree was obtained using the Tree Annotator program in the BEAST package. The final chronogram and node ages were visualised in FigTree v1.4.3 [53]. In addition, the lineages through time (LTT) [54] analysis was conducted to determine the tempo of the species diversification and to assess its possible relation to climatic changes and geological events. LTT plots of the log numbers of the lineages against the log divergence time were constructed using the packages ape and ggplot2 in R v.3.2.

### 2.5. Ancestral Area Reconstructions

Ancestral area reconstructions (AARs) were conducted using RASP 4.2 [55] with the statistical dispersal-vicariance method (S-DIVA) [56] and dispersal-extinction cladogenesis (S-DEC) method [57]. The S-DEC model allows for sympatric speciation, allopatric speciation, and anagenetic range expansion and contraction. Considering the rapid radiation of *Parnassius*, the subsequent analysis was mainly based on the S-DEC model. For the AARs based on topography, we divided the geological area distribution of *Parnassius* into six regions as follows: QTP and Xinjiang; Mid-Eastern China, Korea, and Japan; Northeast Asia; North America; Central and Western Asia; and Europe. The time trees used in this analysis were generated through BEAST v1.83. The plot of AAR was realised by obtaining the marginal probabilities of alternative ancestral distributions integrated with the statistical dispersal-vicariance analysis frequencies of an ancestral range at a node averaged for all trees.

## 3. Results

### 3.1. Sequence Alignment and Genetic Distances

The DNA sequence alignments of the mitochondrial genes *ND1*, *ND5*, and *COI* of 45 *Parnassius* species in this study were 453, 750, and 600 bp, respectively. None of the concatenated DNA sequences contained indels or stop codons. These fragments contained 643 variable sites, of which 515 were Parsimony-informative sites. The interspecific genetic distances estimated among the 45 *Parnassius* species showed that the lowest genetic distance (0.0038) was between *P. phoebus* (Fabricius, 1793) and *P. bremeri* Bremer, 1864, and the highest distance (0.0907) was between *P. hardwickii* Gray, 1831 and *P. tenedius* Eversmann, 1851 (Table S3).

**Figure 2:**
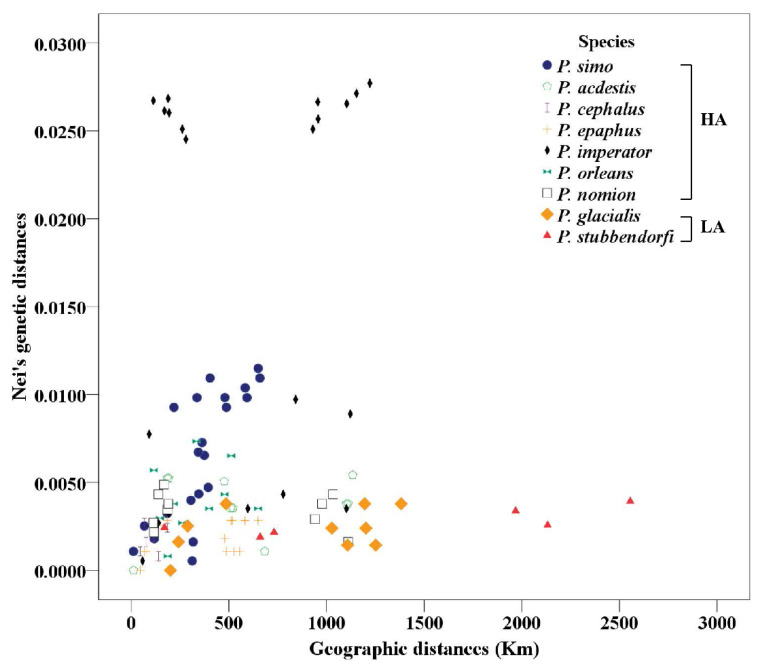
Genetic distance versus geographic distance among the populations of *Parnassius* species in this study. HA: high-altitude; LA: low-altitude. The altitude distributions of the *Parnassius* species in this study are presented in Table 2.

Among the newly sequenced 239 imagoes of 12 *Parnassius* species, 93 mitochondrial haplotypes were detected (Table S1). The genetic and geographic distances were analysed among the populations of these *Parnassius* species with at least three populations (Figure 2 and Table 1), and the results showed that significant differences exist between low- and high-altitude species (Table 2). For the low-altitude *Parnassius* species, *P. stubbendorfii* Ménétriés, 1849 covers a mean geographic distance of 1370 km and a mean genetic distance of 0.0027; *P. glacialis* covers a mean geographic distance of 839 km and a mean genetic distance of 0.0023. However, the high-altitude species, namely *P. imperator*, *P. simo*, and *P. orleans*, cover relatively shorter mean geographic distances of 593, 360, and 330 km, corresponding to relatively greater mean genetic distances of 0.0171, 0.0065, and 0.0041, respectively. For the geographic distribution patterns, our previous study of 13 *P. glacialis* populations supported the isolation-by-distance (IBD) hypothesis of low-altitude species through a Mantel test [17]; in this study, we found a similar IBD pattern in seven populations of the high-altitude species *P. simo* (*p* = 0.01).

### 3.2. Phylogenetic Analysis and Divergence Times

**Figure 3:**
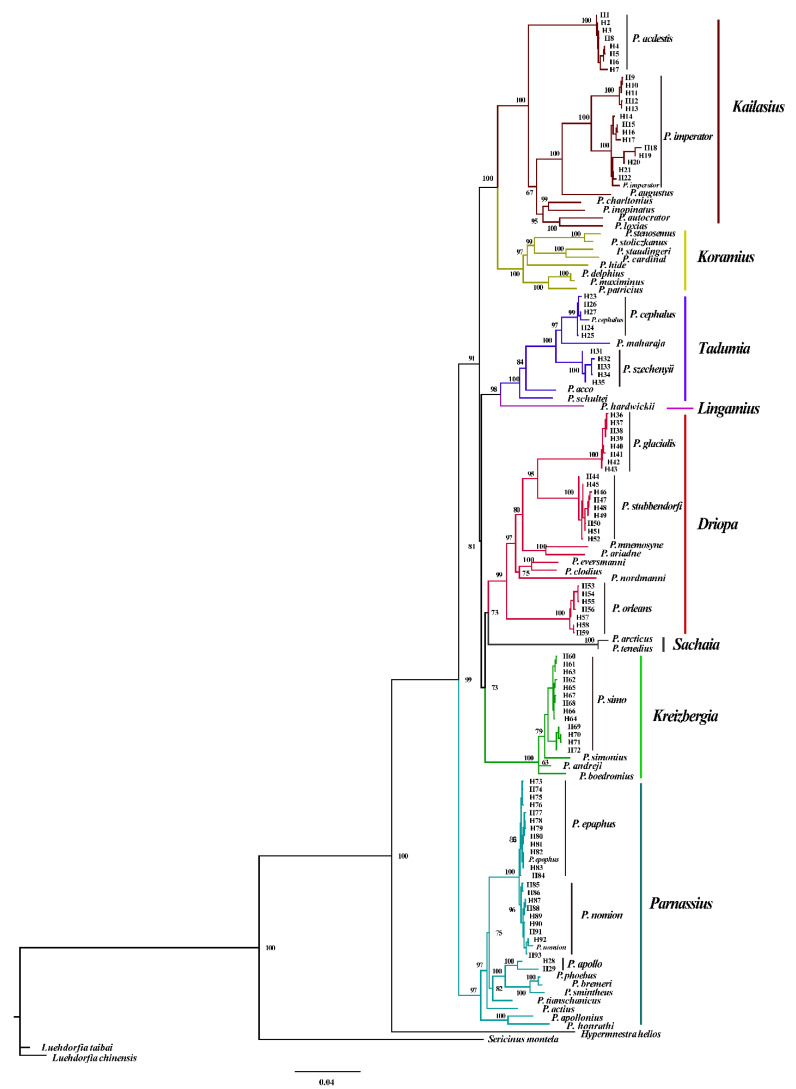
Maximum likelihood phylogenetic tree of 45 *Parnassius* species based on three mitochondrial gene sequences. The sequences are shown in File S1.

**Figure 4:**
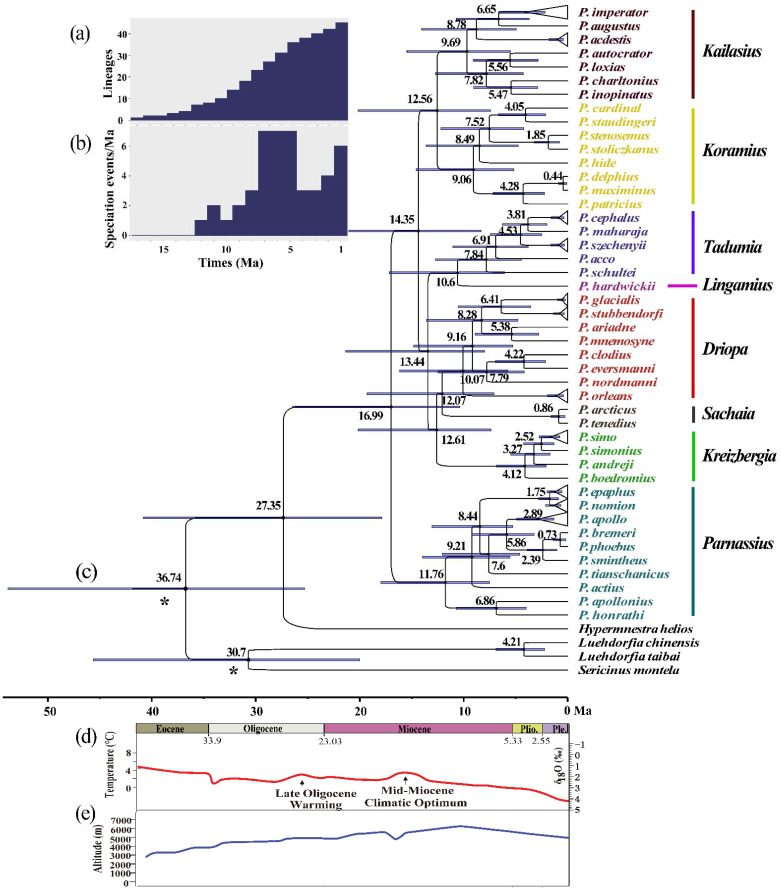
Estimated divergence dates and diversification rate of *Parnassius* and their association with geological and climatic events. (**a**) Lineage-through-time plot and 95% confidence intervals of lineage diversification. (**b**) Diversification rate per million years since the Early Miocene. The dashed line represents the rapid diversification events of *Parnassius*. (**c**) Divergence time estimates through BEAST, with 95% HPD intervals at the branches. * Indicates the calibration points. (**d**) Global temperature curve based on oxygen isotopes [4]. (**e**) Elevation curve of the Qinghai–Tibetan Plateau (QTP) since the Eocene [59].

The phylogenetic analyses inferred using the BI and ML methods resulted in virtually identical tree topologies with high supporting values for most clades (Figure 3). The *Parnassius* species are grouped into eight major subgenera: *Parnassius*, *Tadumia*, *Sachaia*, *Lingamius*, *Kreizbergia*, *Driopa*, *Kailasius*, and *Koramius*. Among these, *Driopa* and *Sachaia* form a clade, sister to *Kreizbergia*; this three-subgenera clade clusters with the *Tadumia* + *Lingamius* group, and this five-subgenera group then clusters with *Kailasius* + *Koramius* (Figure 3). On the basis of this cladogram, the divergence times were estimated, showing that *Parnassius* diverged approximately 16.99 Ma (95% HPD and 26.45–10.40 Ma during the Late Eocene to Middle Miocene; Figure 4c,d), with subgenera *Parnassius* and *Driopa* each diverging at approximately 11.76 Ma (95% HPD, 17.98–7.51 Ma) and 10.07 Ma (95% HPD, 16.17–5.74 Ma), respectively. The other four subgenera (*Tadumia*, *Kailasius*, *Koramius*, and *Kreizbergia*) distributed in the QTP and Central and Western Asia began to diverge at approximately 9.69–4.12 Ma (Late Miocene to Early Pliocene). Our time tree (Figure 4) indicated that the population expansions of *Parnassius* butterflies occurred during the Late Miocene cooling (7–5.4 Ma) [58] and Quaternary Glaciation periods (since 2.58 Ma).

**Figure 5:**
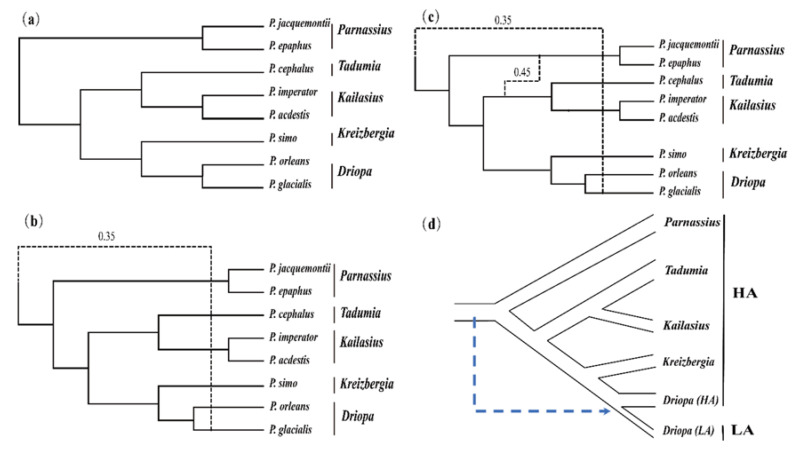
Phylogenetic networks inferred through PhyloNet by using maximum pseudo-likelihood. (**a**) Zero reticulation model. (**b**) One reticulation model. (**c**) Two reticulation model. (**d**) Hypothetical introgression event causing LA divergence. Decimals in figures (**b**,**c**) are the estimated percentage likelihoods of ancient introgressions with 1896 single-copy unigenes.

As shown in Figure 5, our network analyses of the 1896 single-copy unigenes (File S2) identified among *S. montelus* and eight *Parnassius* species, with the 0~2 reticulation models inferred using PhyloNet with the maximum pseudo-likelihood method, indicate that at least two ancient introgression events occurred among the *Parnassius* clades: (1) ancient introgression from the common ancestor to *P. glacialis* (Figure 5b), likely responsible for the diversification of *P. glacialis* from high-altitude species and their spread to low-altitude areas of Eastern China (Figure 5d), and (2) ancient gene introgression from the ancestors of subgenera *Tadumia* and *Kailasius* to the subgenus *Parnassius* (Figure 5c), reflecting frequent gene flows among closely related clades distributed in high-altitude mountains during the early speciation of *Parnassius*.

### 3.3. Ancestral Area Reconstruction

**Figure 6:**
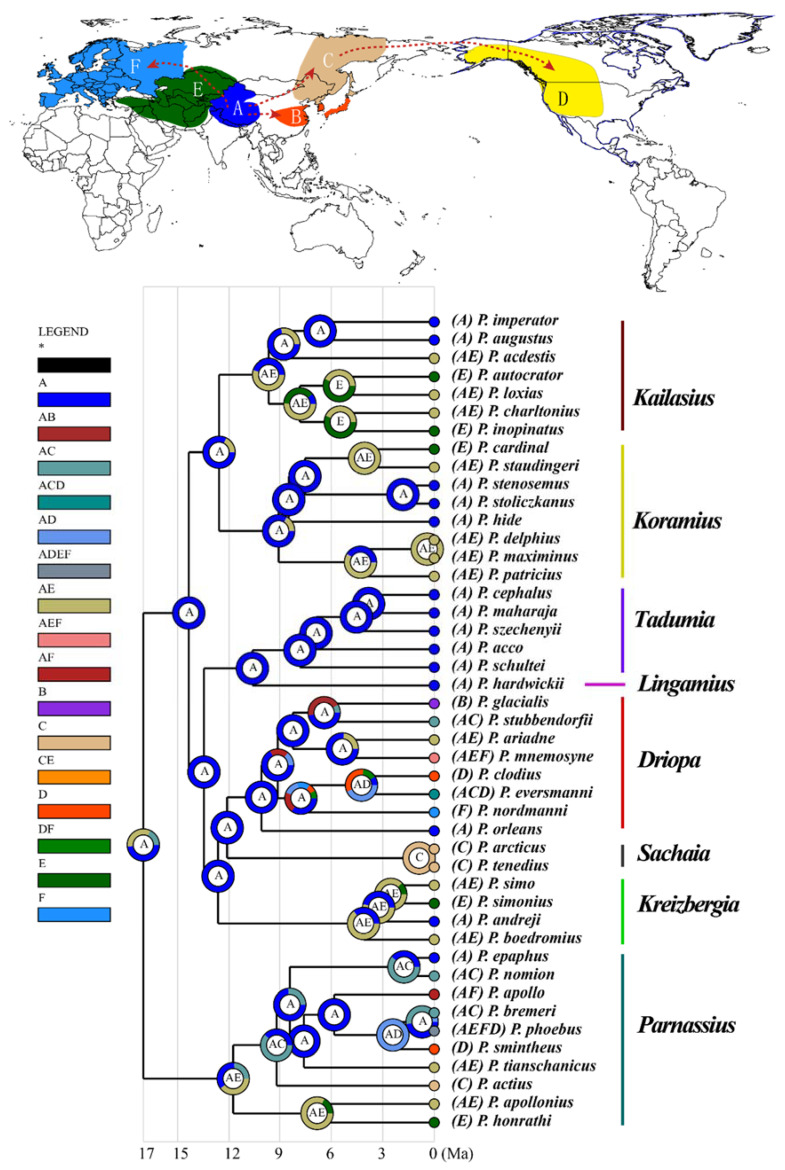
Time tree and ancestral area reconstruction of *Parnassius* based on mitochondrial DNA. Ancestral area assignments at the nodes represent the marginal probabilities of alternative ancestral distributions obtained through statistical dispersal–extinction cladogenesis: (A) QTP and Xinjiang; (B) Central East China, Korea, and Japan; (C) Northeast Asia; (D) North America; (E) Central and Western Asia; and (F) Europe. * Indicates all regions.

Our AAR of the S-DIVA model (Figure S1) showed a similar origination (QTP and/or Central Asia) as previous studies [28]. Unlike S-DIVA, the S-DEC model indicated that *Parnassius* originated in the regions of West China (QTP and Xinjiang) during the Middle Miocene (Figure 6). The historical biogeography of the subgenus *Parnassius* is closely related to the evolution of its host plants (Crassulaceae), such as *Rhodiola*, which originated in the QTP and spread to all of Eurasia by rapid radiation from 12 Ma [60]. Our study indicated that the subgenus *Parnassius* was undergoing speciation during this time (Figure 6). Moreover, our reconstruction shows that the common ancestor of other subgenera (*Tadumia*, *Sachaia*, *Lingamius*, *Kreizbergia*, *Driopa*, *Kailasius*, and *Koramius*) originated in the QTP region (Figure 6A) and then gradually spread to other regions of Eurasia and North America, which is supported by the fact that the speciation of these subgenera is largely consistent with the phylogeographic history of their host plants belonging to the Saxifragaceae family [61].

The empirical LTT plot (Figure 4a) showed that the diversification rate of *Parnassius* increased significantly during the Late Miocene. Our reconstructed speciation rate curve (Figure 4b) showed that *Parnassius* experienced a remarkable increase in species diversification during the Late Miocene and Early Pleistocene (approximately 7.0–5.0 and 2.0–1.0 Ma, respectively).

## 4. Discussion

Studies have shown that the QTP and its neighbouring Asian regions experienced five main stages of uplift associated with climatic events [62]: the India–Eurasia collision (55–40 Ma), the early uplift of the QTP (45–35 Ma), the extension of uplift and the onset of the monsoon system in Asia (35–20 Ma), the progressive uplift of high mountain ranges and aridification of Central Asia (20–10 Ma), and the final extension of the uplift (10 Ma–present). These Cenozoic events triggered the rapid radiation of animals and plants through habitat fragmentation, the key factor leading to the formation of morphologically and physiologically novel habitats and interspecific hybridisation [60,63,64]. Our study indicates that the genus *Parnassius* originated approximately 20–10 Ma (Middle Miocene) during the aforementioned progressive uplift of the mountain ranges and aridification in Central Asia (Figure 4).

Due to rapid diversification driven by environmental changes, some inconsistencies exist in the *Parnassius* phylogeny based on mitochondrial or nuclear genes [12,13,25,26,27,28,65]. Most of them place the subgenus *Parnassius* at the basal position and cluster the other subgenera into three clusters: *Driopa* + *Kreizbergia*, *Tadumia* + *Lingamius*, and *Kailasius* + *Koramius*. Although mitochondrial genes might affect the phylogenetic stability caused by the peculiarities of inheritance, our results (Figure 3), which are based on three mitochondrial genes, are consistent with the phylogeny generated by multiple mitochondrial and nuclear genes (*ND1*, *ND5*, *16S*, *COI*, and *EF-1α*) in a previous study [28]. A few studies have placed the subgenus *Driopa* at the basal position [12], which is contrary to the results of this study. Our network analysis suggests that an ancient introgression probably occurred from the hypothetical common ancestor to generate *P. glacialis* (Figure 5b); the consequent gene flows would have reduced the genetic distance between the common ancestor and the subgenus *Driopa*, causing difficulties in its phylogenetic reconstruction. A similar ancient gene introgression is suggested between the ancestor of the subgenera *Tadumia* plus *Kailasius* and the basal subgenus *Parnassius* (Figure 5c). These gene introgressions are probably caused by frequent geographical overlaps of closely related taxa during the rapid topographic and climatic changes in the region, similar to that found in *Heliconius* butterflies, causing difficulties in its phylogenetic inference [66,67].

Studies have shown that plants belonging to the Crassulaceae family are the primary host plants for the subgenus *Parnassius*, whereas other subgenera of the genus *Parnassius* are associated with Papaveraceae or Saxifragaceae [14,68]. Butterflies have been found to coevolve with their host plants over the course of evolutionary history [60,69]. Our divergence time estimates (Figure 4) indicate that *Parnassius* butterflies began to diverge during the Early Miocene, approximately 16.99 Ma, during a phase of rapid uplift of the QTP (Figure 4e) associated with extensive mountain building in the region, which likely caused habitat fragmentation, leading to subsequent allopatric speciation, which is considered the most essential factor driving the early rapid diversification of *Parnassius*. Along with climate cooling after the Middle Miocene Climate Optimum (MMCO, ~17–14 Ma) [4] and during the Quaternary Ice Age (2.6–0.1 Ma), *Parnassius* butterflies broke the barriers of mountains and valleys and spread out from the QTP and adjacent Central Asia (Figure 4 and Figure 6). These distributional changes correlated with the host plants of *Parnassius* butterflies—that is, *Rhodiola* (Crassulaceae), as shown in previous studies [60], originated in the QTP and diversified during the Middle Miocene, whereas another family of host plants (Saxifragaceae) spread from Eastern Asia to Western Asia and Northeast Asia [69]. Moreover, the expansion process of *Parnassius* butterflies has been proven by previous studies on single-species phylogeography (for example, *P. apollo* (Linnaeus, 1758) [18,70], *P. phoebus* [71], and *P. glacialis* [17]), although these species might show different patterns of expansion and contraction caused by glacial–interglacial cycles during the Quaternary period.

Our AAR (Figure 6) indicates that most *Parnassius* butterflies were distributed in Asia and Europe during earlier evolutionary periods, whereas a few species, such as *P. clodius* Ménétriés, 1855, *P. eversmanni* Ménétriés 1850, *P. smintheus* Doubleday, 1847, and *P. phoebus*, later extended their distribution to North America. Among the subgenus *Driopa*, *P. nordmanni* Ménétriés, 1850 and *P. mnemosyne* (Linnaeus, 1758) reached Europe at approximately 7.8 and 5.4 Ma (Late Miocene) (Figure 4 and Figure 6), respectively, probably through the intervening mountain ranges in Central Asia. Furthermore, another migration group, including *P. clodius* and *P. eversmanni*, reached the mountain ranges in North China, such as the Qinling Mountains and Taihang Mountains, and further dispersed towards North America. The members of the subgenus *Parnassius*, namely *P. apollo*, *P. sminthius*, and *P. phoebus*, show similar routes of dispersion to Europe and North America during the Quaternary Ice Age (Figure 4 and Figure 6), reflecting the distributional history of their host plant: *Rhodiola* (Crassulaceae) [60].

Additionally, we found that *P. glacialis* extended its distribution from the high mountains of the QTP to the low-altitude areas of Central East China (Figure 6), possibly also during the Quaternary Ice Age. Our results indicate that, compared with the high-altitude species, the relatively lower-altitude populations of *P. glacialis* harboured significantly lower genetic distances versus geographic distances (Figure 2); their drastically different new habitats have led to remarkable morphological adaptations, such as body size enlargement and wing colour lightening. We suggest that probable ancient gene introgression events, as shown in our network analysis (Figure 5b,d), might have contributed to the adaptive evolution of *P. glacialis*.

## 5. Conclusions

Our analyses of 239 specimens of 12 *Parnassius* species, collected from the QTP and neighbouring regions, show that the eight-subgenus phylogeny was resolved based on three mitochondrial gene (*COI*, *ND1*, and *ND5*) sequences, with subgenus *Parnassius* at the basal position; the crown-group of genus *Parnassius* originated during the Middle Miocene (ca. 16.99 Ma), coeval with the rapid uplift phase of the QTP and extensive orogeny in the regions of West China and Central Asia. Ancestral area reconstruction of the *Parnassius* species indicates that, during the progressive climate cooling after MMCO, dispersal likely occurred from West China (QTP and Xinjiang) to Central Asia, East and North China, Europe, and North America. We found that the early diversification and biogeographic changes of *Parnassius* are also associated with the butterflies’ host plants in time and space. We conducted phylogenetic network analyses based on 1896 single-copy unigenes (File S2) identified among eight *Parnassius* species, which suggested that ancient gene introgression events probably occurred during the rapid radiation of *Parnassius* due to the geographical overlaps of closely related taxa and interspecific hybridisation. In addition, we found that some low-altitude species, such as *P. glacialis*, harbour a significantly lower interspecific genetic divergence against geographic distance among populations compared with alpine populations, suggesting a higher rate of gene flow among low-latitude butterflies than among high mountain butterflies.

## Figures and Tables

**Table 1:** General information regarding the mitochondrial sequences of 45 *Parnassius* and 4 outgroup species. Details of the samples collected for this study are presented in Table S1.

Species	*ND1* (Locality)	*COI* (Locality)	*ND5* (Locality)
*Hypermnestra helios* Nickerl, 1846	AJ972131 (Uzbekistan)	AM231506 (Uzbekistan)	AB095659 (Uzbekistan)
*Sericinus montela* Gray, 1853	AJ972136 (Fuchu, Japan)	AF170868 (Fuchu, Japan)	AB095665 (Kyoto, Japan)
*Luehdorfia chinensis* Leech, 1893	EU622524 (NA)	EU622524 (NA)	EU622524 (NA)
*Luehdorfia taibai* Chou, 1994	KC952673 (NA)	KC952673 (NA)	KC952673 (NA)
*P. tianschanicus* Oberthür, 1879	DQ407806 (Dolon Pass, Kyrgyzstan)	DQ407767 (Dolon Pass, Kyrgyzstan)	AB095648 (Alai, Kyrgyzstan)
*P. phoebus* Fabricius, 1793	AJ972122 (Tomtor, Yakutia, Russia)	AM231499 (Tomtor, Yakutia, Russia)	AB095654 (Magadan, Russia)
*P. honrathi* Staudinger, 1882	AJ972129 (Ghissarski Mts, Uzbekistan)	DQ407772 (Ghissarski Mts, Uzbekistan)	AB096091 (Uzbekistan)
*P. schultei* Weiss & Michel, 1989	AJ972073 (Tibet, China)	AM231445 (Tibet, China)	AB095619 (Tibet, China)
*P. smintheus* Doubleday, 1847	AJ972125 (Wyoming, USA)	AM231495 (Wyoming, USA)	AB095653 (Colorado, USA)
*P. maharaja* Avinoff, 1916	AJ972076 (Ladakh, India)	AM231448 (Ladakh, India)	AB095615 (Ladakh, India)
*P. acco* Gray, 1852	AJ972070 (Ladakh, India)	AM231442 (Ladakh, India)	AB095652 (Tibet, China)
*P. mnemosyne* Linnaeus, 1758	AM283061 (Kyrgyzstan)	AM231422 (Kyrgyzstan)	AB095626 (Kyrgyzstan)
*P. eversmanni* Ménétriés, 1850	AJ972056 (Amur, Russia)	AM231430 (Amur, Russia)	AB095608 (Amur, Russia)
*P. andreji* Eisner, 1930	AJ972068 (Sichuan, China)	AM231440 (Sichuan, China)	AB095643 (Sichuan, China)
*P. stenosemus* Honrath, 1890	AJ972089 (Zanskar, India)	AM231461 (Zanskar, India)	AB095656 (Ladakh, India)
*P. simonius* Staudinger, 1889	DQ407809 (Kyrgyzstan)	DQ407758 (Kyrgyzstan)	AB095649 (Kyrgyzstan)
*P. charltonius* Gray, 1852	AJ972079 (Kyrgyzstan)	AM231451 (Kyrgyzstan)	AB095630 (Kyrgyzstan)
*P. hardwickii* Gray, 1831	AJ972069 (E. Nepal)	DQ407770 (E. Nepal)	AB094969 (E. Nepal)
*P. clodius* Ménétriés, 1855	AJ972058 (California, USA)	AF170871 (California, USA)	AB095624 (Montana, USA)
*P. staudingeri* Bang-Haas, 1882	AJ972103 (Kaltakol, W. Gissar, Uzbekistan)	AM231477 (Kaltakol, W. Gissar, Uzbekistan)	AM283087 (Kaltakol, W. Gissar, Uzbekistan)
*P. nordmanni* Ménétriés, 1850	AJ972059 (Caucasus, Russia)	AM231432 (Caucasus, Russia)	AB094968 (Caucasus, Russia)
*P. autocrator* Avinoff, 1913	AJ972082 (Tajikistan)	AM231454 (Tajikistan)	AB095634 (Tajikistan)
*P. loxias* Püngeler, 1901	AJ972080 (Kyrgyzstan)	AM231452 (Kyrgyzstan)	AB096090 (Kyrgyzstan)
*P. delphius* Eversmann, 1843	AJ972092 (Kyrgyzstan)	DQ407762 (Kyrgyzstan)	AB095632 (Kyrgyzstan)
*P. inopinatus* Kotzsch, 1940	AJ972081 (Afghanistan)	AM231453 (Afghanistan)	AB095641 (Afghanistan)
*P. patricius* Niepelt, 1911	AJ972091 (Kyrgyzstan)	AM231463 (Kyrgyzstan)	AB095620 (Tianshan, Xinjiang, China)
*P. boedromius* Püngeler, 1901	AJ972067 (Kyrgyzstan)	AM231439 (Kyrgyzstan)	AB095629 (Tianshan, Xinjiang, China)
*P. hide* Koiwaya, 1987	AJ972090 (Tibet, China)	AM231462 (Tibet, China)	AB095613 (Qinghai, China)
*P. ariadne* Lederer, 1853	AJ972055 (Altai, Russia)	AM231429 (Altai, Russia)	AB094970 (Altai, Russia)
*P. stoliczkanus* Felder & Felder, 1864	AJ972087 (Ladakh, India)	AM231459 (Ladakh, India)	AB095650 (Ladakh, India)
*P. arcticus* Eisner, 1968	AJ972062 (Yakutia, Russia)	AM231434 (Yakutia, Russia)	AB095639 (Yakutia, Russia)
*P. maximinus* Staudinger, 1891	AJ972094 (Tianshan, Xinjiang, China)	AM231466 (Tianshan, Xinjiang, China)	AB095651 (Tianshan, Xinjiang, China)
*P. cardinal* Grum-Grshimailo, 1887	AJ972095 (Tajikistan)	AM231467 (Tajikistan)	AB095644 (Tajikistan)
*P. tenedius* Eversmann, 1851	AJ972063 (Yakutia, Russia)	AM231435 (Yakutia, Russia)	AB095658 (Yakutia, Russia)
*P. actius* Eversmann, 1843	DQ407807 (Tianshan, Xinjiang, China)	DQ407765 (Tianshan, Xinjiang, China)	AB095622 (Tianshan, Xinjiang, China)
*P. bremeri* Bremer, 1864	AJ972126 (Korea)	AM231501 (Korea)	AB095611 (Korea)
*P. augustus* Frühstörfer, 1903	AJ972084 (Tibet, China)	AM231456 (Tibet, China)	AB095645 (Tibet, China)
*P. imperator* Oberthür, 1883	AJ972083 (Qilianshan, Gansu, China)	DQ407775 (Qilianshan, Gansu, China)	AB095612 (Qinghai, China)
*P. cephalus* Grum-Grshimailo, 1891	AJ972075 (Kun Lun Shan, China)	AM231447 (Kun Lun Shan, China)	AB095616 (Qamdo, Tibet, China)
*P. epaphus* Oberthür, 1879	AJ972104 (Hankar, Ladakh, India)	AM231478 (Hankar, Ladakh, India)	AB095610 (Qilianshan, Gansu, China)
*P. nomion* Fischer de Waldheim, 1823	AJ972109 (Datong Shan, Qinghai, China)	AM231480 (Datong Shan, Qinghai, China)	AB095609 (Primorye, Russia)
*P. imperator* Oberthür, 1883	This study (7 populations)	This study (7 populations)	This study (7 populations)
*P. cephalus* Grum-Grshimailo, 1891	This study (4 populations)	This study (4 populations)	This study (4 populations)
*P. epaphus* Oberthür, 1879	This study (6 populations)	This study (6 populations)	This study (6 populations)
*P. nomion* Fischer de Waldheim, 1823	This study (5 populations)	This study (5 populations)	This study (5 populations)
*P. acdestis* Grum-Grshimailo, 1891	This study (5 populations)	This study (5 populations)	This study (5 populations)
*P. szechenyii* Frivaldszky, 1886	This study (2 populations)	This study (2 populations)	This study (2 populations)
*P. glacialis* Butler, 1866	This study (5 populations)	This study (5 populations)	This study (5 populations)
*P. stubbendorfii* Ménétriés, 1849	This study (4 populations)	This study (4 populations)	This study (4 populations)
*P. orleans* Oberthür, 1890	This study (5 populations)	This study (5 populations)	This study (5 populations)
*P. simo* Gray, 1852	This study (7 populations)	This study (7 populations)	This study (7 populations)
*P. apollo* Linnaeus, 1758	This study (2 populations)	This study (2 populations)	This study (2 populations)
*P. apollonius* Eversmann, 1847	This study (1 population)	This study (1 population)	This study (1 populations)

**Table 2:** Mean intraspecific geographic and Nei’s genetic distances of the *Parnassius* species.

Species	Mean Genetic Distance	Mean Geographic Distance (km)	Main Altitude Distribution (m)
*P. glacialis*	0.0023	839	200–2000
*P. stubbendorfii*	0.0027	1370	300–2500
*P. nomion*	0.0034	498	2000–3500
*P. imperator*	0.0171	593	2800–5100
*P. orleans*	0.0041	330	3000–5000
*P. epaphus*	0.0020	368	3800–5100
*P. acdestis*	0.0037	593	4000–5000
*P. simo*	0.0065	360	4000–5100
*P. cephalus*	0.0041	193	4000–5100

## Data Availability

The mitochondrial gene sequences of this study were deposited into GenBank with accession numbers MH518317–MH520060. The transcriptomic sequencing data was downloaded from the GenBank BioProject (PRJNA591246).

## Supplementary Materials

The following supporting information can be downloaded at https://www.mdpi.com/article/10.3390/insects13050406/s1: Figure S1: Ancestral area reconstruction obtained by the statistical dispersal-vicariance method. Table S1: Sample information of 12 *Parnassius* species and their populations used in this study. Table S2: List of primers used for PCR amplification. Table S3: Kimura 2-Parameter genetic distance among the 45 *Parnassius* species in this study. File S1: One hundred and thirty-four concatenated mitochondrial segments (*COI*, *ND1*, and *ND5*) of 45 *Parnassius* and 4 outgroup species. File S2: One thousand, eight hundred and ninety-six single-copy unigene sequences identified among *S. montelus* and 8 *Parnassius* species. oob: *P. orleans*; jbo: *P. jacquemontii*; agg: *P. acdestis*; iob: *P. imperator*; cgr: *P. cephalus*; sgr: *P. simo*; ess: *P. epaphus*; bqj: *P. glacialis*; sdf: *S. montelus*.
