# Supplementary material for: Phylogeny and Biogeographic History of Parnassius Butterflies (Papilionidae: Parnassiinae) Reveal Their Origin and Deep Diversification in West China
Source: Insects. 2022 Apr 23;13(5):406. doi: 10.3390/insects13050406 (PMC9142892; doi:10.3390/insects13050406)

Supplementary Figures:

Figure S1: Ancestral area reconstruction obtained by statistical dispersal-vicariance method. (A) QTP and Xinjiang; (B) Central East China, Korea and Japan; (C) Northeast Asia; (D) North America; (E) Central and Western Asia; (F) Europe.

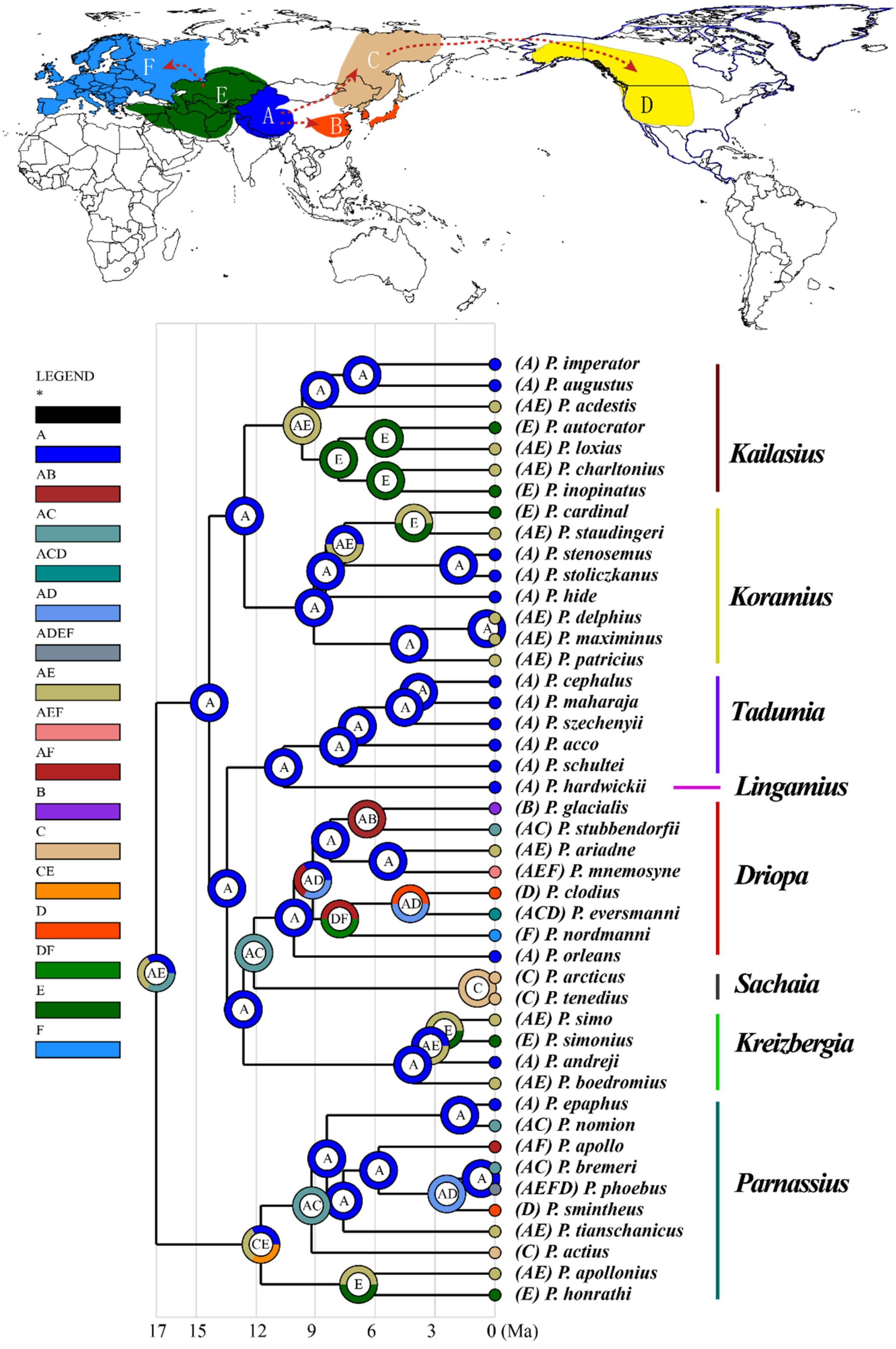

Supplement: Supplementary file 1 [file insects-13-00406-s001.zip › Supplementary-Figure-S1.pdf]
